# Low rate of non-attenders to primary care providers in Israel - a retrospective longitudinal study

**DOI:** 10.1186/2045-4015-3-15

**Published:** 2014-04-25

**Authors:** Dana Rosen, Sasson Nakar, Arnon D Cohen, Shlomo Vinker

**Affiliations:** 1 Department of Family Medicine, Sackler School of Medicine, Tel-Aviv University, Tel-Aviv, Israel; 2 Department of Family Medicine, Central District, Clalit Health Services, Rehovot, Israel; 3 Siaal Research Center for Family Practice and Primary Care, Faculty of Health Sciences, Ben-Gurion University of the Negev, Beer-Sheva, Israel; 4 Chief Physician Office, headquarters, Clalit Health Services, 17/2 Yehoshua Bin-Nun St., Hod Hasharon, Tel Aviv 45236, Israel

**Keywords:** Primary care physician, Non-attender, Health services, Preventive services

## Abstract

**Background:**

A model that combines reactive and anticipatory care within routine consultations has become recognized as a cost-effective means of providing preventive health care, challenging the need of the periodic health examination. As such, opportunistic screening may be preferable to organized screening. Provision of comprehensive preventive healthcare within the primary care system depends on regular attendance of the general population to primary care physicians (PCPs). Objectives: To assess the proportion of patients who do not visit a PCP even once during a four-year period, and to describe the characteristics of this population.

**Methods:**

An observational study, based on electronic medical records of 421,012 individuals who were members of one district of Clalit Health Services, the largest health maintenance organization in Israel.

**Results:**

The average annual number of visits to PCPs was 7.6 ± 8.7 to 8.3 ± 9.0 (median 5, 25%-75% interval 1–11) and 9.5 ± 10.0 to10.2 ± 10.4 (median 6, 25%-75% interval 1–14) including visits to direct access consultants) in the four years of the study. During the first year of the study 87.2% of the population visited a PCP. During the four year study period, only 1.5% did not visit a PCP even once.

In a multivariate analysis having fewer chronic diseases (for each additional chronic disease the OR, 95% CI was 0.40 (0.38¬0.42)), being a new immigrant (OR, 95% CI 2.46 (2.32¬2.62)), and being male (OR, 95% CI 1.66 (1.58¬1.75)) were the strongest predictors of being a non-attender to a PCP for four consecutive years.

**Conclusions:**

The rate of nonattendance to PCPs in Israel is low. Other than new immigrant status, none of the characteristics identified for nonattendance suggest increased need for healthcare services.

## Background

This work was performed in partial fulfillment of the M.D. thesis requirements of the Sackler Faculty of Medicine, Tel Aviv University.

Debate continues over the contribution to preventive healthcare of the periodic health examination (PHE, also known as the general health check and the routine physical examination) [1,2]. The Canadian Task Force recommended its abandonment (Canadian Task Force 1979) decades ago and the United States Preventive Services Task Force (USPSTF) issued patient specific recommendations for preventive healthcare instead of a standard general health check (USPSTF 1997). Higher rates of screening and diagnoses have been reported among populations who undergo PHEs [3-5]. However, if the same persons who undergo PHEs also regularly visit primary care physicians (PCPs), then regular physician visits, whether to PCPs or to PHEs, may be central to optimizing preventive healthcare services. A model that combines reactive and anticipatory care within routine consultations in the primary medical care setting has in fact become recognized as a cost-effective means of providing preventive health care, challenging the need of the PHE [6-8]. As such, opportunistic screening may be preferable to organized screening.

Krogsbøll et al. conducted a Cochrane review of randomized control studies about general health checks for reducing illness and mortality published until 2010 [9]. Their conclusion was that with the large number of participants and deaths included, the long follow-up periods used in the trials, and considering that death from cardiovascular diseases and cancer were not reduced, general health checks are unlikely to be beneficial.

Provision of comprehensive preventive healthcare within the primary care system depends on regular attendance of the general population to PCPs, particularly by members of subpopulations who are in most need of preventive healthcare services.

We conducted an observational study based on the electronic medical records of the largest health maintenance organization (HMO) in Israel to investigate the proportion and characteristics of patients who do not regularly visit PCPs.

## Methods

### Population and data source

Data were retrieved from the Clalit Health Services (CHS) central computerized database. CHS insures and provides healthcare to 54% of the Israeli population (above 4,000,000 people). For over a decade, records of all the visits to PCPs, as well as to consultants in the community, have been fully computerized, and the information is accessible from a central repository. The database includes demographic characteristics, information about physician visits and a register of a selected number of chronic diseases [10].

The population of this study consists of all people of all ages who were members of CHS Central District in 01/01/2007 and remain members during the entire four year period 2007–2010. Individuals who left the HMO or the district during this period were excluded. Patients who died during the study years were included. The final study population included 421,012 of 481,474 CHS Central District members at January 2007.

### Data accessed

The number of visits of CHS members to a PCP and direct access consultations (DACs) was retrieved for each of the four years of the study period. Additional patient data included: *Demographic characteristics:* age, gender, country of birth, year of immigration to Israel (Individuals who were born in Ethiopia and immigrated to Israel after 1984 (the first large wave of immigration from Ethiopia to Israel) were defined as “new immigrants”. Immigrants from other countries were defined as “new immigrants” if they immigrated after 1990 (The year that started a large wave of immigration from the USSR to Israel)), residency (urban or rural), socioeconomic status (SES; low SES was defined as exemption from social security payments only due to low income); and *Chronic disease diagnoses,* as retrieved from the central chronic diseases registry of CHS: malignancy, diabetes, hyperlipidemia, drug abuse, dementia, epilepsy, ischemic heart disease (IHD), congestive heart failure (CHF), hypertension, cerebro-vascular accident (CVA), chronic obstructive pulmonary disease (COPD), and asthma. The number of chronic diseases at the beginning of 2007 was summed for each individual. A diagnosis of anxiety disorder was retrieved as well.

Five medical specialties in the Israeli community health system provide healthcare by DAC, i.e. do not require a letter of referral from a PCP: Ear Nose and Throat (Otorhinolaryngology, ENT), Gynecology, Orthopedics, Ophthalmology, and Dermatology. For consultation with all other community physicians, as well as at all hospital outpatient clinics, a referral letter from a PCP is required. The documentation of visits to DACs is similar to the visits to PCPs.

Main outcomes: The number of non-attenders to primary care service was assessed: for the year 2007, for the combined two years 2007–2008, for 2007–2009, and finally for the four years 2007 to 2010. The denominator (total population) decreased in each consecutive year by the number of people who died in the previous year.

The study was approved by the CHS ethics committee at the Meir Medical Center.

### Statistical analysis

Descriptive statistics was the primary method of analyzing the data in this population study. The dependent variable was visit frequency, dichotomized to any number of visits vs. no visits. Demographic characteristics were compared as well as medical characteristics for sub-groups according to visit frequency, using chi-squared analysis and T-tests.

We used multivariate analysis to construct predictive models for comparison between patients who did not visit a PCP during the four year study period and the rest of the study population. A multivariate logistic regression model was applied to the data to study simultaneously the independent relationship between the demographic (age, gender, SES, residence area, and immigration status) and clinical background (number of chronic diseases) and the main outcome. The model predicts the probability of being a non-attender to a PCP as a function of the explanatory variables.

A p-value of 0.05 or less was considered statistically significant. All analyses were carried out using SPSS ver.18 statistical software.

## Results

Table 1 shows the characteristics of the study population. Most of the population (64%) had no chronic diseases and 25% had one or two chronic diseases. The frequencies of visits of the study population to PCPs, and to PCPs and DACs, for each of the years in the study period, 2007–2010 are presented in Table 1. The average annual number of visits to PCPs was 7.6 ± 8.7 to 8.3 ± 9.0 (median 5, 25%-75% interval 1–11) and 9.5 ± 10.0 to10.2 ± 10.4 (median 6, 25%-75% interval 1–14) including visits to direct access consultants) in the four years of the study.

**Table 1:** Characteristics of study population and visits to primary care physicians and direct access consultants

**Characteristic**		**N (%) (unless stated otherwise)**
**N**		421,012 (100)
**Gender**	Male	202,071 (48)
**Age (in yrs)**	Mean (±SD)	37.1 (23.7)
	Median	35
	0-4	30,488 (7.2)
	5-14	69,296 (16.5)
	15-19	22,893 (5.4)
	20-29	56,830 (13.5)
	30-44	73,400 (17.4)
	45-54	54,881 (13.0)
	55-64	50,944 (12.1)
	65-74	32,189 (7.6)
	75+	30,091 (7.1)
**Socioeconomic status**	Others	368,331 (87.5)
	Low SES	52,681 (12.5)
Place of residence*	Large city (≥100,000 citizens)	144,848 (34.4)
	Other city	230,465 (54.8)
	Rural	45,699 (10.8)
**Country of birth**	Born in Israel	277,426 (65.9)
	Year of immigration < 1990	90,150 (21.4)
	New immigrants	53,436 (12.7)
**Chronic diseases (number)**	Mean (±SD)	0.72 (1.30)
**Anxiety**	Yes	10,598 (2.5)
**Number of visits in 2007 (Mean ± SD, median, 25%-75% interval) n = 421,012**	Primary care	7.6 (8.7) (5, 2–10
	Primary care & direct access consultants**	9.5 (10.0) (6, 2–13)
Number of visits in 2008 (Mean ± SD, (median, 25%-75% interval) n = 418,091*	Primary care	7.9 (8.8) (5, 2–10)
	Primary care & direct access consultants**	9.8 (10.1) (7, 3–14)
Number of visits in 2009 (Mean ± SD, (median, 25%-75% interval) n = 415,024*	Primary care	8.3 (8.9) (6, 2–10)
	Primary care & direct access consultants**	10.2 (10.2) (7, 3–14)
Number of visits in 2010 (Mean ± SD, (median, 25%-75% interval) n = 412,012*	Primary care	8.3 (9.0) (6, 2–10)
	Primary care & direct access consultants**	10.2 (10.4) (7, 3–14)

*For the years 2008–2009, n does not include individuals who died during the previous year(s) of the study period.

**Direct access visits comprise the five medical specialties in the Israeli community health system that do not require a letter of referral from a family physician: Ear Nose and Throat (ENT), Gynecology, Orthopedics, Ophthalmology, and Dermatology.

Tables 2 and 3 present the numbers and percentages of individuals who did not visit a PCP, and who did not visit a PCP or DAC, respectively, during a 1, 2, 3, and 4 year period, according to demographic and clinical characteristics. For all the characteristics analyzed, statistically significant differences (P < 0.001) were observed in nonattendance to PCPs (Table 3) and to PCPs or DACs (Table 3). While 12.8% of the study population did not visit a PCP during a single year (2007), only 1.5% did not visit a PCP even once during a four year period. Among young children (ages 0–4) and older adults (ages 55–74), the percentages of non-attenders to the PCP were lower than among other age groups. The subgroup with low SES had a relatively low proportion of non-attenders. Altogether, 73.5% of the population visited a PCP at least once each year and 79.2% visited either a PCP or a direct access consultant (DAC) every year during the four year study period. More males were non-attenders than females. Of males aged 20–44, 3% did not visit a PCP in the four year study period and 2.3% did not visit a PCP or a DAC. In contrast, only 1.2% of women of childbearing age did not visit a PCP during the four year study period, and only 0.9% did not visit a PCP or a DAC.

**Table 2:** Non-attenders to primary care during a 4 year period

		**Did not visit during one year (2007)**	**Did not visit during 2 years (2007–2008)**	**Did not visit during 3 years (2007–2009)**	**Did not visit during 4 years (2007–2010)**
**Total cohort**		421,012	418,091	415,024	412,012
**Total n (%)**		54,093 (12.8)	21,264 (5.1)	10,391 (2.5)	6,217 (1.5)
Gender n (%*)	Male	31,074 (15.4)	12,606 (6.3)	6,418 (3.2)	3,733 (1.9)
	Female	23,019 (10.5)	8,658 (4.0)	3,973 (1.9)	2,484 (1.2)
	p Value	<0.001	<0.001	<0.001	<0.001
Age group in 2007 n (%*)	0–4	1,492 (4.9)	557 (1.8)	291 (1.0)	214 (0.7)
	5–14	11,933 (17.2)	4,566 (6.6)	1,931 (2.8)	1,149 (1.7)
	15–19	4,598 (20.1)	2,432 (10.6)	1,281 (5.6)	551 (2.4)
	20–29	9,807 (17.3)	3,813 (6.7)	1,948 (3.4)	1,160 (2.0)
	30–44	12,222 (16.7)	4,823 (6.6)	2,508 (3.4)	1,596 (2.2)
	45–54	6,620 (12.1)	2,356 (4.3)	1,127 (2.1)	678 (1.2)
	55–64	3,972 (7.8)	1,452 (2.9)	676 (1.4)	402 (0.8)
	65–74	1,417 (4.4)	421 (1.4)	240 (0.8)	166 (0.6)
	75+	2,032 (6.8)	844 (3.2)	389 (1.6)	301 (1.3)
	p Value	<0.001	<0.001	<0.001	<0.001
Socioeconomic status n (%*)	Others	50,705 (13.8)	20,004 (5.5)	9,718 (2.7)	5,828 (1.6)
	Low SES	3,388 (6.4)	1,260 (2.5)	673 (1.4)	389 (0.8)
Residence n (%*)	Large city**	17,998 (12.4)	7,235 (5.1)	3,910 (2.8)	2,546 (1.8)
	Other cities	25,844 (11.9)	10,243 (4.5)	5,179 (2.3)	3,040 (1.4)
	Rural	10,251 (22.4)	3,786 (8.3)	1,302 (2.8)	631 (1.4)
	p Value	<0.001	<0.001	<0.001	<0.001
Country of birth n (%*)	Born in Israel	39,942 (14.6)	15,262 (5.6)	6,981 (2.5)	3,785 (1.3)
	Year of immigration ≤1990	6,360 (7.2)	2,327 (2.8)	1,057 (1.3)	667 (0.8)
	New Immigrants	7,791 (13.9)	3,675 (6.7)	2,353 (4.4)	1,765 (3.3)
	p Value	<0.001	<0.001	<0.001	<0.001
**Sum of chronic diseases**	**Mean (±sd)**	0.24 (0.73)	0.18 (0.36)	0.16 (0.59)	0.17 (0.62)
Anxiety n (%*)	No	53,755 (13.1)	21,138 (5.2)	10,351 (2.6)	6,194 (1.6)
	Yes	338 (3.2)	126 (1.2)	40 (0.4)	23 (0.2)
	p Value	<0.001	<0.001	<0.001	<0.001

*Percentage within group of that characteristic.

**Large cities – ≥100,000 citizens.

**Table 3:** Non-attenders to primary care or direct access consultants during a 4 year period

		**Did not visit during one year (2007)**	**Did not visit during 2 years (2007–2008)**	**Did not visit during 3 years (2007–2009)**	**Did not visit during 4 years (2007–2010)**
**Total cohort**		421,012	418,091	415,024	412,012
**Total n (%)**		39,247 (9.3)	14,944 (3.6)	7,713 (1.9)	4,793 (1.2)
Sex n (%*)	Male	24,453 (12.1)	94,25 (4.7)	49,39 (2.5)	29,08 (1.5)
	Female	14,794 (6.8)	5,519 (2.6)	2,774 (1.3)	1,885 (0.9)
	p Value	<0.001	<0.001	<0.001	<0.001
Age group in 2007 n (%*)	0–4	1,265 (4.1)	449 (1.5)	253 (0.8)	195 (0.6)
	5–14	9,408 (13.6)	3,326 (4.8)	1,485 (2.1)	920 (1.3)
	15–19	3,473 (15.2)	1,765 (7.7)	933 (4.1)	403 (1.8)
	20–29	6,721 (11.8)	2,594 (4.6)	1,386 (2.4)	841 (1.5)
	30–44	8,550 (11.6)	3,326 (4.5)	1,834 (2.5)	1,217 (1.7)
	45–54	4,816 (8.8)	1,685 (3.1)	848 (1.6)	549 (1.0)
	55–64	2,863 (5.6)	1,034 (2.1)	502 (1.0)	312 (0.6)
	65–74	917 (2.8)	315 (1.0)	202 (0.7)	146 (0.5)
	75+	1,234 (4.1)	450 (1.7)	270 (1.1)	210 (0.9)
	p Value	<0.001	<0.001	<0.001	<0.001
Socioeconomic status n (%*)	Others	36,612 (9.9)	13,978 (3.8)	7,212 (2.0)	4,502 (1.2)
	Low SES	2,635 (5.0)	966 (1.9)	501 (1.0)	291 (0.6)
	p Value	<0.001	<0.001	<0.001	<0.001
Residence n (%*)	Large city**	12,934 (8.9)	4,987 (3.5)	2,923 (2.1)	1,977 (1.4)
	Other cities	19,978 (8.7)	7,757 (3.4)	3,948 (1.7)	2,388 (1.1)
	Rural	6,335 (13.9)	2,200 (4.8)	842 (1.8)	428 (0.9)
	p Value	<0.001	<0.001	<0.001	<0.001
Country of birth n (%*)	Born in Israel	28,731 (10.5)	10,413 (3.8)	4,920 (1.8)	2,722 (1.2)
	Year of immigration ≤1990	4,227 (4.8)	1,497 (1.8)	762 (0.9)	496 (0.6)
	New immigrants	6,289 (11.2)	2,734 (5.0)	2,031 (3.7)	1,575 (2.9)
	p Value	<0.001	<0.001	<0.001	<0.001
**Sum of chronic diseases**	**Mean (±sd)**	0.21 (0.68)	0.15 (0.54)	0.14 (0.54)	0.15 (0.56)
Anxiety n (%*)	No	39,041 (9.5)	14,874 (3.7)	7,678 (1.9)	4,773 (1.2)
	Yes	206 (1.9)	70 (0.7)	35 (0.3)	20 (0.2)
	p Value	<0.001	<0.001	<0.001	<0.001

*Percentage within group of that characteristic.

**Large cities – >100,000 citizens.

Table 4 presents a multivariate logistic regression analysis for those who did not visit a PCP during the four year study period, compared to the rest of the study population. Only those who were alive for the entire four-year period were included in this analysis. All the variables that were statistically significant in the univariate analysis were found to be associated with nonattendance during a four year period in the multivariate analysis. Having fewer chronic diseases, being a new immigrant, and being male were the strongest predictors of being a non-attender to a PCP for four consecutive years.

**Table 4:** Subgroups of non-attenders to a PCP during a four year period (multivariate logistic regression)

**Characteristic**	**OR (95% C.I.)**	**p value**
N chronic diseases (for each additional chronic disease)	0.40 (0.38-0.42)	<0.001
“New immigrant” vs. Israeli born	2.46 (2.32-2.62)	<0.001
Male vs. Female	1.66 (1.58-1.75)	<0.001
SES (not low vs. low)	1.41 (1.27-1.57)	<0.001
Age group (in comparison to age group 65–74)		
0–4	1.16 (0.94-1.42)	0.16
5–14	2.80 (2.37-3.03)	<0.001
15–19	4.30 (3.61-5.13)	<0.001
20–29	3.38 (2.86-3.99)	<0.001
30–44	3.64 (3.10-4.28)	<0.001
45–54	2.11 (1.77-2.50)	<0.001
55–64	1.35 (1.30-1.62)	<0.001
65–74	1.0	
75+	2.51 (20.7-3.03)	<0.001
Rural vs. non-rural residence	1.09 (1.01-1.18)	0.039

## Discussion

During a four year period, 73.5% of the general population visited a primary care physician (PCP) at least once a year, and 79.2% visited either a PCP or a direct access consultant (DAC) every year. Only 1.5% did not visit a PCP during four consecutive years, and 1.2% visited neither a PCP nor a DAC during a four-year period. It follows that the great majority of individuals visited physicians at a frequency that would enable the provision of preventive care according to current Israeli preventive task force recommendations [11]; during acute or chronic care visits. Further, very young children and older adults were age groups least likely to be non-attenders to a PCP, and thus available for case finding preventive care. This is consistent with recommendations for the provision of preventive healthcare services for pregnant women, the very young, and the very old [12].

Surveys show that both patients and physicians expect and value annual visits to a PCP [13-15]. Moreover, individuals who receive annual examinations were found to feel better, behave healthier, undergo more appropriate screening, and trust their physicians more than patients who did not have annual examinations [13,16]. Recently, Medicare enrollees who did not visit a primary care physician during a one year period were reported to be less likely to be tested for colorectal cancer than those who did visit [17]. Still, the benefit of PHEs in real patient outcomes: morbidity and mortality have not been established [9]. Whether chronic and acute care visits can provide the same quality of healthcare and physician-patient relationship as a dedicated PHE visit has yet to be determined.

The rate of individuals who did not visit a PCP during three years, 2.5%, is lower than the 9.7% rate reported from a study of 32 general practitioner practices in England [18]. In the National Health Insurance system in Israel all residents are covered; and a visit to a PCP in the CHS is free of copayment. These two facts may contribute to the high visit rate observed in the current study.

The main characteristics of non-attenders to a PCP were: male, age 15 to 44 years, not with low SES, having few or no chronic diseases and being a new immigrant. Other than new immigrant status, none of the characteristics found to be prominent among non-attenders correspond with higher health risks than in the rest of the population. This supports information gained from interviews with three-year non-attenders to general practitioner services in England [18]. The investigators of the study recommended against targeting the non-attenders for preventive healthcare, since their higher levels of perceived health reduced the likelihood of their responding to an invitation to a general health check. From a review of 39 studies, Dryden (2012) reported non-attenders to general checks to be more likely of low socioeconomic status, unemployed or less well educated, and more likely to be single and non-white. Cardiovascular risk factors, including smoking, were more prevalent among non-attenders. Non-attenders valued health less strongly, felt less in control of their health, and were less likely to believe in the efficacy of health checks [19]. In Israel Eshel et al. found that as a group, the older Israelis who do not initiate PCP visits are healthier than those who do [20]. On the other hand Khanassov et al. in a commentary to this study emphasize the fact that the lower rate of chronic disease may be an artifact [21]. The provision of preventive healthcare by means of PCP visits may respond better to population-wide healthcare needs, while PHEs may perpetuate the inverse care law, by which good healthcare is provided inversely to medical need [22].

The demonstration in the current study that the primary care system reaches the vast majority of individuals supports the combination of reactive and anticipatory care within routine consultations as a comprehensive and egalitarian way of providing preventive healthcare, at least in Israel. The exception is the sub-population of new immigrants. Similarly, new immigrants in Canada were found to screen less for cervical cancer than the rest of the population [23].

The capability of PCPs to provide preventive health services is dependent on the time allotted such services as well as physician and patient awareness and cooperation. A retrospective chart audit conducted in Canada revealed that preventive screening occurred at low rates at general practitioner clinics [24]. Not surprisingly, time constraints have been found to limit physician compliance to preventive service recommendations [25,26].

Possible information bias is a limitation of this study. There is a substantial number of physician visits that are administrative (repeat prescription, fill out forms, etc.), and do not necessarily involve actual patient-physician contact, but we presume that in such cases at least some of the annual visits are “face to face” medical encounters. In rural villages, medical problems may more likely be resolved by visits with local nurses, who may be more accessible than physicians. Further, the fact that non-attenders were diagnosed with fewer chronic diseases does not indicate that they are healthier. Nevertheless, non-attenders to the PCP in Israel were found to feel healthier and to be healthier as well [27]. Another limitation to the current study is that some of those classified as “new immigrants”, particularly non-attenders, may live outside Israel. The fact that new immigrants to Israel usually keep their original residency increases the probability of such events.

## Conclusion

In conclusion, we found that in a large cohort, served by the major HMO in Israel, the vast majority (98.5%) of the population visits a physician within a four-year period. Other than new immigrant status, none of the characteristics identified for nonattendance suggest increased need for healthcare services.

These findings support the possibility that implementation of preventive healthcare measures based on the case-finding method in primary care facilities could be practical.

## Competing interests

The authors declare that they have no competing interests.

## Authors’ contributions

DR, SN, ADC and SV have made substantial contributions to conception and design, or acquisition of data, or analysis and interpretation of data; DR, ADC and SV have been involved in drafting the manuscript or revising it critically for important intellectual content; DR, ADC and SV have given final approval of the version to be published; and DR, ADC and SV agree to be accountable for all aspects of the work in ensuring that questions related to the accuracy or integrity of any part of the work are appropriately investigated and resolved. Each author should have participated sufficiently in the work to take public responsibility for appropriate portions of the content. All authors read and approved the final manuscript.

## Authors’ information

Dana Rosen MD thesis at the Tel Aviv University.

In memory of Dr. Sasson Nakar who was one of the tutors of D. Rosen in her Thesis.
